# Analytical and Clinical Validation of Action PharmaKitDx: A Comprehensive NGS Panel for the Identification of Pharmacogenetic Variants in Diverse Populations

**DOI:** 10.3390/ph19040568

**Published:** 2026-04-01

**Authors:** Luis Ramudo-Cela, Marta Izquierdo-García, María Dolores-Sequedo, Vicente Cubells-Perez, Sara Bernal, Pau Riera, Adriana Lasa, Laura Torres-Juan, Victor José Asensio, Iciar Martínez-López, Antonia Obrador de Hevia, Matías Morín, Miguel Ángel Moreno-Pelayo, Greta Carmona-Antoñanzas, Javier Porta Pelayo

**Affiliations:** 1 Health in Code, 46024 Valencia, Spain; marta.izquierdo@healthincode.com (M.I.-G.); loli.sequedo@healthincode.com (M.D.-S.); vicente.cubells@genycell.com (V.C.-P.); greta.carmona@healthincode.com (G.C.-A.); javier.porta@genologica.com (J.P.P.); 2 Hospital Pharmacy Research Group, A Coruña Biomedical Research Institute (INIBIC), A Coruña University Hospital Complex (CHUAC), Galician Health Service (SERGAS), University of A Coruña (UDC), 15006 A Coruña, Spain; 3 Genetics Department, Hospital de la Santa Creu i Sant Pau, 08025 Barcelona, Spain; sbernal@santpau.cat (S.B.); alasa@santpau.cat (A.L.); 4 Institut de Recerca Sant Pau (IR SANTPAU), 08041 Barcelona, Spain; priera@santpau.cat; 5 Pharmacy Department, Hospital de la Santa Creu i Sant Pau, 08025 Barcelona, Spain; 6 Genomics of Health Research Group, Health Research Institute of the Balearic Islands (IdISBa), 07120 Palma, Spain; laura.torresjuan@ssib.es (L.T.-J.); victor.asensio@ssib.es (V.J.A.); iciar.martinez@ssib.es (I.M.-L.); antonia.obrador@ssib.es (A.O.d.H.); 7 Molecular Diagnostics and Clinical Genetics Unit, Son Espases University Hospital, 07120 Palma, Spain; 8 Group of Advanced Therapies and Biomarkers in Clinical Oncology, Institut d’Investigació Sanitària de Palma (IdISPa), 07120 Palma, Spain; 9 Genetics Service, Instituto Ramón y Cajal de Investigación Sanitaria (IRYCIS), Hospital Ramón y Cajal, 28034 Madrid, Spain; matmorinro@yahoo.es (M.M.);; 10 Biomedical Network Research Centre on Rare Diseases (CIBERER), 28029 Madrid, Spain

**Keywords:** pharmacogenomics, next-generation sequencing, clinical pharmacogenetics, drug metabolism, precision medicine, clinical validation, analytical validation, cytochrome P-450 enzyme system, copy number variation

## Abstract

**Background/Objectives**: Pharmacogenomics (PGx) enables personalized therapy by identifying genetic variants that influence drug response. Despite the advantages of next-generation sequencing (NGS), few clinically validated, guideline-aligned panels comprehensively detect common, rare, and structurally complex pharmacogenetic variants. **Methods**: We developed and analytically validated Action PharmaKitDx, a targeted NGS panel covering 335 pharmacogenes, including all priority genes recommended by CPIC, DPWG, and CPNDS. Performance was assessed using Coriell HapMap and GeT-RM reference materials across multiple library preparation workflows and Illumina platforms. Clinical feasibility was evaluated in 41 patient samples from diverse specialties. Results were compared with established reference methods, including PCR-based assays, STR analysis, Sanger sequencing, and whole-exome sequencing. **Results**: Analytical validation: More than 99% of target bases achieved ≥30× coverage. Analytical accuracy, sensitivity, specificity, and positive predictive value exceeded 99.3%, with repeatability and reproducibility >99.7%. Concordance with GeT-RM haplotypes reached 98% after star-allele harmonization. The panel accurately detected complex variants, including *CYP2D6* copy-number changes and hybrid alleles. Clinical validation: Full concordance with prior genotyping was observed in clinical samples. Beyond the initial testing indication, each sample harbored a mean of six actionable variants (range 2–10). Thirty-six rare (minor allele frequency <1%) potentially actionable variants were additionally identified. **Conclusions**: Action PharmaKitDx demonstrates high analytical performance and broad clinical applicability, supporting its implementation as a scalable solution for comprehensive pharmacogenetic testing and precision prescribing.

## 1. Introduction

Pharmacogenomics (PGx) explores how genetic variation influences drug response and has become a key pillar of personalized medicine. By identifying variants that affect drug metabolism, transport, or targets, PGx enables clinicians to optimize therapies to maximize safety and efficacy [1]. Advances in genomic technologies—most notably next-generation sequencing (NGS)—have accelerated the integration of genomics into clinical practice by enabling comprehensive and cost-effective analysis of multiple drug-related genes simultaneously [2,3]. Unlike traditional genotyping methods such as PCR-based assays or microarrays, NGS allows for the detection of both common and rare variants with high sensitivity and specificity [4]. This is particularly relevant as recent evidence suggests that rare variants in pharmacogenes may be more prevalent than previously recognized and can have significant functional implications, impacting drug efficacy or toxicity in ways not captured by standard panels focused only on frequent alleles [5].

Importantly, NGS also enables the detection of complex structural variants and haplotypes in pharmacogenes with intricate genomic architectures, such as *CYP2D6*. The ability to characterize duplications, deletions, and hybrid alleles is essential for accurate genotype-to-phenotype translation in these loci [6,7,8]. By comparison, whole-exome sequencing (WES) is poorly suited to PGx applications because it often fails to cover key non-coding regulatory variants with known clinical relevance [9,10]. For instance, CYP2C19*17, located in the promoter region, increases gene transcription and leads to ultrarapid metabolism of drugs such as clopidogrel; missing this variant can result in inappropriate dosing and elevated bleeding risk. Similarly, UGT1A1*28 in the promoter affects bilirubin clearance and irinotecan toxicity, and *VKORC1* intronic variants strongly influence warfarin sensitivity. These clinically actionable variants are typically outside the exonic regions targeted by WES, making exome-based approaches incomplete for comprehensive pharmacogenomic profiling. While whole-genome sequencing (WGS) may overcome these limitations by offering uniform coverage across the genome, its high cost and complex data interpretation currently limit its feasibility for routine clinical use. Overall, targeted NGS panels based on hybridization capture strike an optimal balance between analytical depth, clinical relevance, and cost-effectiveness, making them particularly well-suited for PGx testing in real-world settings [11,12].

Despite the technical benefits, there is a notable lack of clinically validated NGS kits specifically designed to meet the requirements of pharmacogenomics laboratories. Many commercial panels do not provide adequate coverage of guideline-recommended variants, lack structural variant detection, or are tailored to research rather than clinical diagnostics. This gap has hindered the broader implementation of PGx in healthcare, despite strong evidence supporting its clinical utility [13]. Large cohort studies and clinical trials have shown that over 95% of individuals carry at least one actionable PGx variant, and that pharmacogenomic-guided prescribing reduces adverse drug reactions and healthcare costs [5,14,15]. There is thus an urgent need for scalable, robust, and clinically integrated PGx platforms that not only adhere to scientifically accepted guidelines but also align with national health authority frameworks and current clinical recommendations to ensure regulatory compliance and real-world applicability.

To address the need for a comprehensive NGS-based pharmacogenomics solution, the Action PharmaKitDx panel was carefully designed to encompass genetic variants relevant to drug response, including those recommended by major clinical guidelines—such as the Clinical Pharmacogenetics Implementation Consortium (CPIC) and the Dutch Pharmacogenetics Working Group (DPWG)—and FDA drug labels [16,17]. The development also ensures full alignment with current Spanish and European regulatory frameworks. It incorporates the pharmacogenomic biomarkers listed in the AEMPS’s official database, which reflects inclusion criteria from the National Health System’s genomic services portfolio approved in June 2023, and adheres to EMA’s Guideline on Good Pharmacogenomic Practice, which mandates robust methodology, validation, and clinical interpretation standards for germline genomic assays. The current version complies with the In Vitro Diagnostic Directive (IVDD) and is CE-marked under this framework. Furthermore, technical and regulatory transition plans are actively underway to ensure full compliance with the updated European In Vitro Diagnostic Medical Devices Regulation (IVDR, EU 2017/746) prior to the expiration of the current transition periods, reinforcing its long-term regulatory robustness. The primary objective of this study was to validate the analytical performance of Action PharmaKitDx—including sensitivity, specificity, repeatability, and reproducibility—and to assess its clinical feasibility for implementation in routine pharmacogenetic testing, in line with these regulatory and clinical integration goals.

## 2. Results

### 2.1. Sequencing Performance

Sequencing performance was assessed across four workflow configurations: manual library preparation on the MiSeq system, automated library preparation on the MiSeq system, and fully automated workflows using either the NextSeq 500/550Dx or the NextSeq 1000/2000 platforms. In all cases, library loading was optimized to ensure sufficient clusters passing filter, enabling coverage of more than 98% of bases at depths exceeding 30×.

In the manual MiSeq workflow, the mean Q30 score reached 82.15%, with an average coverage of 582× and 99.3% of bases covered at greater than 30× depth. The automated MiSeq workflow improved these metrics, yielding a mean Q30 of 93.4%, 89.1% of clusters passing filter, an average coverage of 926×, and 99.4% of bases exceeding 30×.

In the fully automated workflows, the NextSeq 500/550 system generated a mean Q30 of 84.3% and 89.4% clusters passing filter. On the NextSeq 1000/2000 system, sequencing performance included a mean Q30 of 90.9% and a total output of 41.3 Gb. Both NextSeq platforms achieved higher mean sequencing depths—1880× on the NexSeq 500/550 and 1400× on the NextSeq 1000/2000—with more than 99.5% of bases covered at depths greater than 30×.

**Figure A1:**
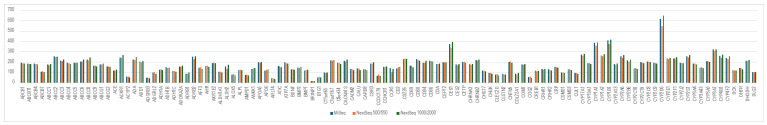

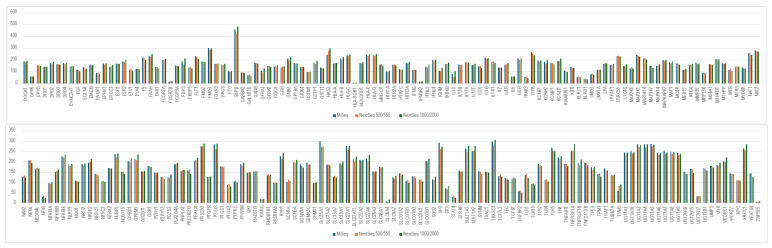
Gene-level mean coverage distribution across the evaluated sequencing workflow. The bar plot illustrates the average sequencing depth achieved for each of the genes included in the Action PharmaKitDx panel. Data are shown for the three sequencing platforms: MiSeq, NextSeq 500/550, and NextSeq 1000/2000. While the vast majority of targets, including highly homologous loci (e.g., *CYP2D6*, *HLA*), demonstrate robust and uniform coverage well above this threshold, a minor subset of highly complex genes (such as *HLA-DQB1* and *SLC6A4*) exhibit mean depths slightly below 30× due to inherent short-read mapping challenges.

To evaluate coverage uniformity across historically challenging genomic regions, a comprehensive gene-by-gene mean coverage analysis was performed across all sequencing workflows. The results are detailed in Appendix B Figure A1, which confirms consistent depth across both standard and complex loci.

Although a direct base-by-base correlation with GC-content was not explicitly modeled, the robust performance observed in highly homologous genomic regions—such as the *CYP2D6* and *HLA* loci—is supported by a targeted, direct haplotype capture probe design. This specialized design, combined with a laboratory protocol optimized to generate sufficiently long DNA fragments and paired-end sequencing read lengths, facilitates accurate read alignment and target discrimination in these highly homologous contexts.

These optimized metrics ensured that sufficient sequencing depth was achieved across most of the targeted regions. It is important to note, however, that a minor subset of targets—specifically *HLA-DQB1*, *ZNF595*, *FCGR2B*, *IFNGR2*, *STAT4*, *SLC6A4*, *RARG*, and *NFIB*—exhibited a mean coverage slightly below the 30× threshold. This drop-off reflects the extreme sequence complexity, repetitive nature, or inherent mapping challenges associated with these specific loci using short-read next-generation sequencing technologies.

### 2.2. Analytical Performance

The analytical performance of the Action PharmaKit panel was evaluated using three well-characterized reference DNA samples (NA12878, NA12891, and NA12892) (Table 1).

Across all workflows, analytical specificity (NPA) and PPV were greater than 99.9%, demonstrating the absence of false-positive variant calls within the panel’s target regions. Analytical accuracy and sensitivity were consistently high, with mean values exceeding 99.3% for all workflows.

In the manual MiSeq workflow, mean analytical accuracy and sensitivity were 99.9% and 99.4%, respectively, with mean repeatability and reproducibility averaging 99.07%. The automated MiSeq workflow achieved optimal performance, achieving 100% across all metrics for all reference samples. In the NextSeq 500/550 platform, mean analytical accuracy and sensitivity were 99.9% and 99.8%, respectively, with repeatability and reproducibility of 99.8%. Similarly, the NextSeq 1000/2000 workflow yielded uniformly high performance across all metrics, with analytical accuracy, sensitivity, repeatability, and reproducibility each reaching 99.9%.

Observed differences in sensitivity and concordance were attributable to variant calls detected by the pipeline that did not meet predefined quality (score > 42) and/or coverage (>30×) thresholds. Detailed counts of true positives (TPs), true negatives (TNs), false positives (FPs), and false negatives (FNs) for each replicate and workflow are provided in Appendix B.2 Table A1. Across all evaluated workflows and replicates, a total of four variants were not correctly reported in at least one replicate. Three of these variants were located in low-complexity regions and one in a GC-rich region, both recognized as challenging contexts for next-generation sequencing.

No false negatives were observed for the NA12878 sample across any workflow. For NA12891, one false negative occurred in a single replicate of the NextSeq 500/550 workflow due to insufficient variant quality. For NA12892, three false negatives were observed in the manual MiSeq workflow due to insufficient coverage. Additionally, the same variant observed as a false negative in NA12891 was also not reported in one replicate of NA12892 in the NextSeq 500/550 platform due to low variant quality. Finally, one additional false negative was observed for NA12892 in the NextSeq 1000/2000 platform due to insufficient coverage, corresponding to one of the variants also affected in the manual workflow.

Importantly, all pharmacogenetically relevant positions included in the panel were consistently detected across workflows and sequencing runs, supporting the robustness of the assay for clinical pharmacogenetic applications.

### 2.3. Pharmacogenetic Haplotypes

To evaluate the accuracy of pharmacogenetic haplotype and genotype calling, the 20 previously described Coriell GeT-RM reference samples were analyzed. Concordance was defined as the proportion of haplotypes identically assigned by the Action PharmaKitDx workflow and the GeT-RM reference dataset. This analysis was performed across 16 pharmacogenes included in the Action PharmaKitDx core panel and characterized by the GeT-RM program: *CYP1A2*, *CYP2B6*, *CYP2C9*, *CYP2C19*, *CYP2D6*, *CYP3A4*, *CYP3A5*, *CYP4F2*, *DPYD*, *NAT2*, *SLCO1B1*, *TPMT*, *UGT1A1*, *VKORC1*, *HLA-A*, and *HLA-B*.

Across 250 allele-level genotype calls, the initial concordance with the GeT-RM reference was 91.2%. Most discrepancies (6.8%) were attributable to differences in the star-allele definitions used by Action PharmaKitDx and the GeT-RM reference materials. Of these, 2.8% occurred because Action PharmaKitDx reported star alleles not defined or represented in the GeT-RM datasets, whereas 4.0% corresponded to alleles present in GeT-RM but not included in the allele database implemented by Action PharmaKitDx.

Because legacy GeT-RM datasets often rely on older nomenclature or partial allele definitions, a standardized harmonization step was performed. The underlying variant calls accurately detected by the pipeline were objectively mapped against the standardized allele definitions. This systematic alignment eliminated nomenclature-driven artifacts without introducing subjective allele reassignments, thereby increasing the overall concordance rate to 98%.

**Figure 1:**
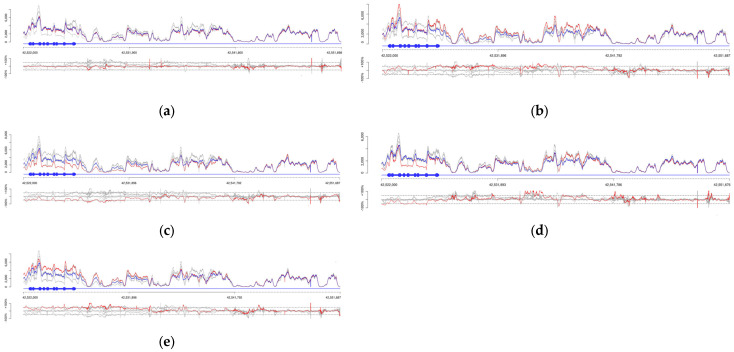
Strand-aware coverage analysis of the CYP2D6 locus and its homologous regions in five GeT-RM reference samples. These samples were selected to represent the main structural variation patterns at the CYP2D6 locus, whose genotypes have been established through community-standard orthogonal methods, including ddPCR and long-read sequencing [18]. Orientation and conventions: The x-axis shows genomic coordinates that increase 5′→3′ along the reference plus (forward) strand. *CYP2D6* is encoded on the reverse (minus) strand, so its transcription proceeds right-to-left in the plot. Blue boxes depict the *CYP2D6* gene structure (thick = exons, thin = introns). Panels and lines: For each sample—NA12892 (**a**), NA12878 (**b**), HG00276 (**c**), HG01190 (**d**), HG00436 (**e**)—the top panel shows absolute read depth (coverage), and the bottom panel shows normalized coverage (adjusted for probe hybridization efficiency). Red lines correspond to the sample under analysis; grey lines are other samples in the sequencing pool; the blue line in the top panel is the internal control (NA17254). Genomic context: Adjacent high-homology blocks (e.g., CYP2D7-derived segments) are included on the x-axis to resolve hybrid and tandem configurations. Sample interpretations (copy-number logic: ~50% decrease = heterozygous loss; ~50% increase = single extra copy): (**a**) NA12892: No deviations in coverage; consistent with diploid *CYP2D6* (two copies; no CNVs). (**b**) NA12878: A focal ~50% coverage gain at the *CYP2D6* 5′ end (exon 1) and the adjacent high-homology region supports a *CYP2D6–CYP2D7* hybrid tandem involving exon 1, consistent with CYP2D6*68, with two *CYP2D6* copies plus the hybrid segment. (**c**) HG00276: A ~50% coverage reduction across the entire CYP2D6 gene indicates a heterozygous deletion (CYP2D6*5), yielding one functional copy. (**d**) HG01190: A ~50% reduction across most of *CYP2D6*, combined with increased coverage in the upstream homologous block, indicates co-occurrence of a heterozygous deletion (*5) and a tandem *68 hybrid. (**e**) HG00436: A uniform ~50% coverage gain across the whole *CYP2D6* region is consistent with a gene duplication (*CYP2D6*×2), totaling three gene copies.

Given the well-known complexity of the *CYP2D6* locus due to frequent structural rearrangements—including gene whole gene deletions (CYP2D6*5), gene duplications (*1×N, *2×N, etc.), and hybrid alleles (*CYP2D6/CYP2D7* and *CYP2D7/CYP2D6* conversions)—an additional concordance analysis was performed using a graphical visualization tool that displays the relative depth of coverage for each *CYP2D6* exon in the test sample compared to an internal control, supporting the identification and classification of duplications and deletions (Figure 1). All known duplications, deletions, and hybrid alleles present in the reference materials were correctly identified and annotated by the pipeline.

### 2.4. Clinical Feasibility

To further evaluate the clinical feasibility of Action PharmaKitDx, a retrospective analysis was performed on 41 patient samples obtained from several participating hospitals. These samples had been previously genotyped using established and validated clinical laboratory methodologies, including allelic discrimination PCR, fluorescent PCR for copy number analysis, short-tandem-repeat (STR) analysis, sanger sequencing, and whole-exome sequencing. The panel demonstrated complete concordance in allele-level distribution (100%) when compared with the reference genotypes generated using these methods. The results derived from this comparative analysis are summarized in Table 2.

Each of the clinical samples included in the study corresponded to a real diagnostic request intended to support therapeutic decision-making in diverse pharmacogenetic contexts, thereby providing a representative collection of use-cases reflective of routine clinical practice. The distribution of clinical indications and associated pharmacogenes is presented in Table 3.

Beyond concordance with targeted clinical requests, analysis of these clinical samples with the NGS panel revealed additional actionable findings across other genes included in Action PharmaKitDx. On average, each sample carried 6 actionable variants (range: 2–10), suggesting a broader potential utility of comprehensive pharmacogenetic profiling compared with single-gene approaches. Moreover, a total of 36 rare potentially actionable variants were identified. Variants were considered “rare” when presenting a minor allele frequency <1% across population databases (gnomAD, 1000G, 5000G and dbSNP) and meeting quality thresholds (depth ≥ 30 and high base quality). Candidate variants were further restricted to those with predicted functional relevance, including non-synonymous coding changes, frameshift or truncating variants, and splicing alterations, supported when applicable by computational predictors (DANN, FATHMM, MutationTaster) or non-benign ClinVar annotations (Table S1). The complete filtering and prioritization strategy applied to identify these variants is described in detail in the Appendix A. This streamlined filtering demonstrates the panel’s ability not only to validate clinically expected variants but also to reveal additional rare alleles with potential clinical significance.

Overall, Action PharmaKitDx demonstrated full concordance with previously established methods across all 41 clinical samples, while simultaneously providing a richer spectrum of pharmacogenomic information. The ability to detect both common guideline-recommended variants and rare alleles underscores its reliability, comprehensiveness, and adds clinical value. These results support its applicability for pharmacogenetic testing across multiple therapeutic domains, including psychiatry, oncology, transplantation, gastroenterology, neurology, and cardiology, and point to its promise as a tool for more holistic pharmacogenomic implementation in clinical practice.

## 3. Discussion

Next-generation sequencing (NGS) has become a transformative technology for pharmacogenetic (PGx) testing, offering capabilities that extend far beyond the constraints of traditional single-nucleotide variant (SNV) genotyping panels. By enabling simultaneous detection of rare and clinically relevant variants, complex structural variants, hybrid alleles, gene deletions or duplications, and full haplotypes, NGS provides a level of genomic resolution that is essential for accurate phenotype prediction and therapeutic decision-making in PGx. This comprehensive variant detection is particularly impactful for pharmacogenes with highly polymorphic architectures—such as CYP2D6, CYP2B6, CYP2C19, and UGT1A1—where conventional methods often fail to resolve complex allele configurations or star-allele diplotypes [11,13].

In this study, the Action PharmaKitDx panel was validated as a targeted NGS solution specifically engineered to address the inherent complexities of pharmacogenomic analysis. The panel integrates broad genomic coverage with high analytical accuracy, enabling robust identification of SNVs, indels, copy-number alterations, gene conversions, and allele-level haplotypes. This design allows reconstruction of clinically actionable star-allele diplotypes across multiple pharmacogenes, ensuring reliable phenotype assignment and enhancing the clinical utility of PGx testing.

The following sections detail the panel’s design, sequencing performance, analytical validation, bioinformatic strategies for haplotype resolution, and the clinical implications of deploying a comprehensive NGS-based approach in routine pharmacogenomic practice, in comparison with previously reported NGS-enabled PGx assays.

### 3.1. Genomic Coverage and Design Strategies

Action PharmaKitDx provides extensive genomic coverage, encompassing 335 drug-related genes organized into 20 primary loci, 11 secondary loci, and 304 candidate loci. This scope exceeds that of most targeted PGx panels currently available, such as ClinPharmSeq and PGRNseq, and is comparable in breadth to the ADME panel described by Klein et al., as summarized in Table 4 [12,19,20]. The inclusion of 304 candidate genes, while not currently dictating CPIC-level prescribing changes, offers immediate clinical value by helping resolve unexplained adverse drug reactions or extreme phenotypes not accounted for by primary pharmacogenes. Furthermore, preemptively sequencing these loci provides the panel with built-in adaptability to future clinical guidelines. As emerging evidence elevates candidate genes to ‘priority’ or ‘secondary’ status, incorporating these updates into routine diagnostics does not necessitate a physical redesign of the hybridization capture probes. Instead, new actionable alleles can be seamlessly integrated by dynamically updating the allele-variant translation tables—derived from PharmVar and PharmGKB—within the automated bioinformatic pipeline, ensuring the assay remains continuously aligned with the latest clinical standards. The design incorporates full coding regions, exon–intron boundaries, selected deep intronic regions, untranslated regions (UTRs), and clinically relevant hotspots across 307 genes. In addition, specialized probes enable direct haplotype identification in complex pharmacogenes such as CYP2D6 and HLA. This hybrid approach resembles that of ClinPharmSeq, which combines an exon-centered design with custom capture of highly polymorphic loci, and that of PGRNseq, which extends sequencing to upstream and downstream UTRs [12,19]. Inclusion of pseudogenes and extragenic regulatory regions is essential, as functionally relevant variants—such as those in CYP2D6 or CYP2C19—can be missed by whole-exome sequencing (WES) [5,21].

### 3.2. Sequencing Performance and Coverage

The panel demonstrated robust sequencing performance, with mean depths of 582× to 1880× depending on the Illumina platform, and >99% of targeted bases covered at ≥30×. These values are comparable or superior to the average coverage depths reported for other targeted approaches (Table 4) [12,19,20]. High and uniform coverage is critical for reliable detection of rare variants and SVs, which are increasingly recognized as clinically relevant in PGx [22].

### 3.3. Analytical Accuracy and Validation

Validation of Action PharmaKitDx showed 100% sensitivity for SNVs and indels, with specificity ranging from 99.99% to 100%. Repeatability and reproducibility exceeded 99.7%. Concordance with GeT-RM reference materials was 98%. The remaining 2% discrepancy reflects inherent limitations of short-read NGS, such as the inability to phase distant variants, which in routine clinical practice would prompt orthogonal confirmation. Notably, the panel correctly identified all known CYP2D6 duplications, deletions, and hybrids—a major challenge in PGx due to the locus’s structural complexity. As summarized in Table 4, the validation metrics of Action PharmaKitDx are highly competitive with those of previously published assays, while offering distinct advantages in broad gene content and comprehensive structural variant capabilities [4,12,19,20]. These findings underscore the analytical robustness of targeted NGS for PGx. A limitation of our haplotype concordance analysis is its restriction to the 16 core pharmacogenes currently characterized by the GeT-RM program. While these include the most structurally complex and clinically urgent loci (e.g., *CYP2D6*, *HLA-A/B*), orthogonal validation strategies will be required to assess haplotype calling accuracy across the broader set of genes. It should be noted, however, that many of the 335 genes included in the panel currently lack established haplotype definitions; for these loci, our analytical validation inherently focused on demonstrating the accurate detection of individual sequence variants (SNVs and indels) rather than full haplotype reconstruction.

### 3.4. Bioinformatics and Clinical Interpretation

The analytical strength of Action PharmaKitDx is further enhanced by its customized bioinformatics pipeline. This includes CYP2D6-specific filtering to distinguish pseudogene alignments (CYP2D7/8), depth normalization, and dedicated modules for HLA-B allele inference [23,24]. Haplotypes are resolved through an in-house algorithm integrating allele–variant translation tables from PharmGKB and PharmVar [25,26]. Comparable pipelines, such as ClinPharmSeq’s use of PyPGx for star allele calling and SV detection, also highlight the importance of tailored bioinformatics in PGx, especially for loci with high structural complexity [12].

### 3.5. Clinical Validation with Patient Samples

A major strength of this study is the validation of the panel using 41 clinical samples derived from real therapeutic contexts across psychiatry, oncology, transplantation, gastroenterology, neurology, and cardiology. Each sample had previously been genotyped using established methods (STR analysis of (TA)n promoter repeat; Sanger sequencing; whole-exome sequencing, allelic-discrimination assays and other PCR-based genotyping methods), enabling direct assessment of concordance. Action PharmaKitDx confirmed all reported genotypes, including clinically relevant star allele combinations and structural variants. Importantly, the panel demonstrated consistent performance across the evaluated clinical scenarios. However, we acknowledge that the relatively small size of our clinical feasibility cohort limits the epidemiological generalizability of these findings. Future multi-center studies involving larger, prospective patient cohorts will be required to comprehensively evaluate the real-world clinical utility and cost-effectiveness of this panel across diverse population demographics

### 3.6. Detection of Rare and Additional Variants

Beyond confirming known variants, the panel revealed additional actionable findings beyond the scope of initial clinical requests. On average, each sample carried 6 actionable variants (range: 2–10), suggesting that a multigene approach may provide broader clinical insights than single-gene testing. This figure is higher than the mean of 3.7 non-typical response pharmacogene alleles per subject reported in the UK Biobank cohort [5] which may reflect differences in panel design, allele classification criteria, and the clinical enrichment of our sample set. Moreover, 36 rare potentially actionable variants were identified in candidate genes, expanding the spectrum of PGx-relevant alleles. This supports previous observations that rare variants account for a substantial fraction of pharmacogenomic diversity and may have significant clinical consequences if functionally characterized [4,13,19,22]. Crucially, because Action PharmaKitDx utilizes a hybridization capture strategy targeting full coding regions and extended boundaries, it is inherently capable of detecting previously unreported alleles. Should novel variants be identified during clinical testing, they can be systematically evaluated and classified using comprehensive bioinformatic workflows. In this study, we applied a multi-step filtering pipeline—detailed in Appendix A.4—that integrates population frequencies, functional impact assessments, and in silico predictors to successfully prioritize 36 rare, potentially actionable variants. This proposed classification strategy can be readily adapted by clinical analysts based on specific diagnostic needs and the availability of bioinformatic tools. From a translational perspective, the clinical reporting of these rare alleles requires a rigorous and cautious framework. Because they currently lack explicit dosing recommendations from CPIC or DPWG, they are generally reported as Variants of Uncertain Significance (VUS) or exploratory findings, accompanied by interpretive disclaimers. In a clinical setting, rather than driving primary prophylactic dose adjustments, these findings serve as highly valuable diagnostic tools to help explain extreme pharmacokinetic phenotypes or severe unresolved adverse drug reactions that cannot be accounted for by traditional star-allele testing.

### 3.7. Clinical Implications and Future Perspectives

Prior studies estimate that >90% of individuals carry at least one clinically actionable PGx variant [5]. Action PharmaKitDx consistently identified reported genotypes in diverse clinical samples—including complex star allele combinations and gene duplications—across therapeutic contexts such as psychiatry, oncology, and immunosuppression. This highlights its potential as a scalable tool for guiding individualized drug therapy.

Accurate detection of actionable variants is foundational for precision medicine. Action PharmaKitDx demonstrated not only 100% concordance with previously established methods across 41 clinical samples, but also an added ability to uncover additional and rare variants. This dual capacity underscores its reliability and broader clinical value compared to traditional genotyping. By consolidating multiple pharmacogenes into a single comprehensive assay, Action PharmaKitDx may streamline workflows, reduce costs, and improve the implementation of PGx-guided prescribing in routine practice. Crucially, the assay is designed to operate seamlessly on standard next-generation sequencing platforms (e.g., MiSeq, NextSeq) and automated library preparation systems that are already widely established in hospital laboratories for routine genetic diagnostics. This compatibility eliminates the need for significant capital expenditure on dedicated, single-purpose equipment, such as microarray scanners. Furthermore, the ability to multiplex up to 32 samples per run, coupled with an estimated sample-to-report turnaround time of approximately 4 to 5 days, significantly reduces per-sample sequencing costs and hands-on time compared to iterative single-gene testing, firmly supporting its scalability and cost-effectiveness for routine clinical use.

Nevertheless, several challenges remain. Short-read NGS cannot easily phase variants separated by long genomic distances. Furthermore, while the accuracy of our *CYP2D6* copy number calling was firmly established using GeT-RM consensus materials—previously characterized by ddPCR and long-read sequencing—and orthogonally validated in clinical samples using fluorescent PCR, a broader assessment is still needed. Extending the use of high-throughput quantitative methods, such as Multiplex Ligation-dependent Probe Amplification (MLPA) or digital PCR (dPCR), to assess structural variants across other pharmacogenes is warranted to fully characterize the panel’s overall CNV detection limits [27,28]. The dynamic nature of allele nomenclature requires continuous alignment with PharmVar and CPIC updates [25]. Moreover, the future integration of long-read sequencing technologies holds significant promise for the evolution of clinical PGx. In the near term, this integration may take the form of a reflex testing strategy, where targeted long-read sequencing is strategically deployed to resolve specific clinical ambiguities—such as unphased distant variants or highly complex, atypical structural rearrangements—that cannot be definitively characterized by the primary short-read assay. Ultimately, adapting our comprehensive target enrichment strategy natively for long-read platforms will allow for the routine direct phasing of all variants into definitive haplotypes and the unambiguous resolution of highly homologous pseudogene boundaries (such as the *CYP2D6*/*CYP2D7* locus), thereby comprehensively overcoming the inherent structural limitations of short-read sequencing [8,29,30].

## 4. Materials and Methods

### 4.1. Panel Design

The panel was conceived to deliver a comprehensive pharmacogenetic assessment fully aligned with clinical practice. Its design integrates a broad set of genes implicated in drug response, integrating them into the standard framework used for pharmacogenetic evaluation. In addition to the primary actionable genes with the highest levels of evidence for predicting drug efficacy, toxicity, or optimal—those prioritized by leading pharmacogenetics guideline-issuing bodies such as the Clinical Pharmacogenetics Implementation Consortium (CPIC), the Dutch Pharmacogenetics Working Group (DPWG), and the Canadian Pharmacogenomics Network for Drug Safety (CPNDS) [16,17]—the panel also incorporates secondary and candidate genes. Although these genes are not yet fully integrated into formal recommendations, accumulating evidence suggests their potential contribution to pharmacokinetic and pharmacodynamic variability, adverse drug reactions, and dose optimization. Their inclusion ensures that the panel remains forward-looking and adaptable to the evolution of evidence in the field, while preserving methodological robustness and scientific validity.

The panel sequences more than 120,000 nucleotides and encompasses 335 genes organized into three evidence-based categories—priority, secondary, and candidate. Priority genes (21 in total), including *ABCG2*, *CACNA1S*, *CYP2B6*, *CYP2C19*, *CYP2C9*, *CYP2D6*, *CYP3A4*, *CYP3A5*, *CYP4F2*, *DPYD*, *F5*, *G6PD*, *HLA-A*, *HLA-B*, *IFNL3*, *NUDT15*, *RYR1*, *SLCO1B1*, *TPMT*, *UGT1A1*, and *VKORC1*, are consistently endorsed by major pharmacogenetic guidelines and represent the highest level of clinical evidence. Secondary genes (10 in total), such as *ABCC3*, *CALU*, *COMT*, *CYP1A2*, *CYP2C18*, *GGCX*, *NAT2*, *RARG*, *SLC28A3*, and *UGT1A6*, are supported by moderate evidence and hold relevance for specific drug–gene interactions. Finally, the candidate category a set of 304 genes currently under active investigation for their potential pharmacogenomic impact, ensuring the panel remains forward-looking and adaptable to emerging evidence.

To ensure comprehensive coverage, the panel combines three complementary sequencing strategies. First, it targets entire coding regions along with intronic boundaries, selected deep intronic segments, and untranslated regions (UTRs) to capture key non-coding variants. Second, it incorporates specialized probe sets for direct haplotype resolution in complex loci like *CYP2D6* and the HLA gene family. Finally, it enriches genomic regions known to harbor clinically relevant variants documented in peer-reviewed literature and curated databases, reinforcing the panel’s clinical utility and evidence-based design. Detailed information on the capture strategies applied to each gene group, as well as the complete list of genes included in the panel, is provided in the Appendix A.

### 4.2. Sample Selection

For the analytical validation of the panel, a total of 20 samples were analyzed. The dataset included 3 reference materials from the Coriell International HapMap Project and 20 reference materials from Coriell’s Pharmacogenetics Genetic Testing Reference Materials Coordination Program (GeT-RM). To ensure robust validation, some samples were sequenced multiple times. Analytical performance evaluation focused on critical quality metrics, including sensitivity, specificity, off-target read assessment, and sequencing performance indicators such as coverage depth and uniformity. Repeatability and reproducibility were rigorously tested through multiple sequencing runs of reference materials under varying conditions.

Clinical performance evaluation was assessed using 41 patient-derived samples from hospitals within the National Public Health System, selected to represent diverse DNA source matrices and sequencing platforms. These samples were selected not to establish epidemiological prevalence, but to represent a diverse array of DNA source matrices, sequencing platforms, and clinically relevant haplotypes across various therapeutic indications. Together, these evaluations provide robust evidence of the panel’s accuracy, reliability, and suitability for routine pharmacogenetic testing.

### 4.3. DNA Extraction

Genomic DNA was isolated at each participating center using extraction workflows routinely implemented in their laboratories. Although the specific extraction methods varied across institutions, all procedures had been previously validated and were regularly employed in accordance with each center’s internal quality assurance and quality control standards. Prior to library preparation, the extracted DNA underwent quantitative assessment and purity evaluation to verify its integrity and ensure compliance with the quality requirements for downstream next-generation sequencing analyses.

Library Preparation and Next-Generation Sequencing

Between 50 and 100 ng of genomic DNA from each sample was processed following the Action PharmaKitDx instructions for use employing the SureSelect XT HS library preparation technology (Agilent Technologies, Santa Clara, CA, USA). The protocol included DNA fragmentation, end-repair, adenylation, and ligation of barcoded adapters. Libraries were purified using Agencourt AMPure XP beads (Beckman Coulter, Brea, CA, USA) and amplified by conventional PCR. The hybridization of the pre-amplified target regions was performed using custom biotinylated probes specifically designed for this study, followed by target capture with streptavidin-coated beads. The captured libraries were subsequently subjected to stringent wash conditions to remove nonspecific hybrids and then re-amplified by PCR to achieve sufficient enrichment. Library quality control included assessment of fragment size distribution between 200 and 250 base pairs, and quantification to confirm that both metrics met the required specifications for sequencing.

Library preparation was performed using both manual and automated workflows. Manual processing was carried out with the [31], whereas automated preparation was completed using the Magnis NGS Prep System (Agilent Technologies) with the CE-marked configuration Action PharmaKitDx (Health in Code, Valencia, Spain). The automated platform is optimized for high-stringency, walk-away processing and enables parallel preparation of up to eight libraries per run, ensuring procedural consistency and reducing operator-dependent variability.

Sequencing was conducted across several Illumina platforms to accommodate different throughput needs. Runs were performed on the MiSeq System sequencer using the Reagent Kit v2 300 cycles (Illumina, San Diego, CA, USA) in batches of 8 samples, on the NextSeq 500/550Dx System using the Mid Output v2.5 300 cycles in batches of 32 samples, and on the NextSeq 1000/2000 using the P2 XLEAP-SBS Reagent Kit 300 cycles, also in batches of 32 samples. Library denaturation and loading procedures were carried out strictly following the manufacturer’s recommendations to ensure optimal cluster generation and sequencing performance.

### 4.4. Bioinformatic Pipeline

Bioinformatic analysis was carried out using a custom pipeline that performs samples demultiplexing and integrates multiple software tools, including NovoAling v4.05, GATK v4.2.6 (Genome Analysis Toolkit), SAMtools v1.15, and BCFtoolds v1.15 for variant calling and genotyping. Initially, raw NGS reads underwent quality-control filtering to remove low-quality sequences. High-quality reads were then aligned to the GRCh37 reference genome. Haplotype inference was performed using a proprietary, deterministic algorithm designed to ensure clinical transparency and reproducibility. Rather than utilizing probabilistic heuristics, the inference engine applies a strict, rule-based approach. It first evaluates the identified variants, their corresponding zygosity, sequence quality metrics, and coverage depth directly from the variant files (VCF format) and coverage data (cov format). The algorithm then systematically compares this sample-specific variant and zygosity profile against a curated database of consensus star-allele definitions derived from the PharmGKB and PharmVar databases [25,26]. By evaluating allele combinations two-by-two, the algorithm requires a perfect match between the variants identified in the sample and the established diplotype combinations in the database to assign the final pharmacogenetic genotype. [32]. Appendix A describe the pharmacogenetic alleles supported by the panel, and the complete list of automatically inferred haplotypes.

*CYP2D6* analysis was performed using a custom-developed module based on the approaches described in [32,33]. This module filters out alignments originating from *CYP2D7* and *CYP2D8* to avoid artifacts in coverage plots caused by miss-mapped reads from highly homologous regions. Following this filtering step, read-depth values are normalized using the control sample NA17254, which reduces biases related to copy-number variation (CNV) events in other samples processed within the same sequencing run.

*HLA-B* allele inference was conducted with a dedicated module implementing the methodology described by Wittig et al. [24], ensuring accurate and reliable *HLA-B* allele assignment for downstream analysis.

## 5. Conclusions

In summary, Action PharmaKitDx delivers a step-change in clinical pharmacogenomics by uniting exceptional genomic breadth with deep, uniform sequencing and purpose-built analytics. Covering 335 drug-related genes with targeted capture of complex loci, the panel reliably detects SNVs, indels, copy-number changes, gene conversions, and allele-level haplotypes, resolving challenging architectures such as *CYP2D6* with a level of fidelity that conventional genotyping and WES routinely miss. Its analytical performance—including 100% sensitivity for SNVs/indels, near-perfect specificity and reproducibility and 98% concordance with reference materials—coupled with accurate calling of *CYP2D6* duplications, deletions, and hybrids, underpins confident genotype-to-phenotype translation. In real-world clinical samples across multiple specialties, Action PharmaKitDx not only confirmed previously reported diplotypes but also revealed additional actionable variants (on average ~6 per case), expanding therapeutic options and informing safer, more individualized pharmacotherapy. Supported by a custom bioinformatics pipeline (*CYP2D6*-aware alignment and depth normalization, *HLA-B* inference, and PharmGKB/PharmVar-anchored haplotyping), the platform delivers end-to-end interpretability aligned with contemporary PGx guidance. Compared with legacy panels and WES—and without the cost and operational burden of WGS—Action PharmaKitDx offers immediate clinical and operational value: it streamlines multigene PGx testing into a single, scalable assay, shortens time-to-insight, reduces downstream costs, and differentiates in the pharmacogenetics market through superior coverage of structural and rare variation. Taken together, these attributes position Action PharmaKitDx as a highly capable, implementation ready tool for the broad deployment of PGx in routine care.

## Figures and Tables

**Table 1:** Sequencing quality metrics and analytical performance of the Action PharmaKitDx panel across workflows.

Parameters		MiSeq (Manual Workflow)	MiSeq (Automated Workflow)	NextSeq 500/550 (Automated Workflow)	NextSeq 1000/2000 (Automated Workflow)
Sequencing quality metrics	Q30 (%)	82.2	93.4	84.3	90.9
	Clusters passing filter (%)	82.2	89.1	89.4	NA
	Mean coverage (×)	582	926	1880	1400
	Bases > 30× (%)	99.3	99.4	>99.5	>99.5
Analytical performance metrics	Analytical accuracy (%)	99.9	>99.9	99.9	99.9
	Analytical sensitivity (PPA%)	99.4	>99.9	99.8	99.9
	Analytical specificity (NPA%)	>99.9	>99.9	>99.9	>99.9
	Positive predictive value (PPV%)	>99.9	>99.9	>99.9	>99.9
	Repeatability (%)	99.1	>99.9	99.8	99.9
	Reproducibility (%)	99.1	>99.9	99.8	99.9

Analytical performance values represent mean results per workflow. NA, not applicable.

**Table 2:** Pharmacogenetic alleles validated in the clinical cohort.

Gene	Detected Allele(s)	Sample ID(s)	Reference Method(s)
*CYP2D6*	*3, *4, *5, *6, *9, *10, *17, *41, *xN*	23V15551, 23T15552, 23Y15555, 23Q15562, 23Z15563, 23P15568, 23R15570, 23V11743	Allelic discrimination PCR; fluorescent PCR for copy number determination; Sanger sequencing
*CYP2C19*	*2, *17	23R15553, 23Y15555, 23X15564, 23P15568, 23R15570, 23Y11750	Allelic discrimination PCR
*CYP2C9*	*2, *3	23P15554, 23Y15555, 23R15570, 23R11745, 23Z11738, 23Q11740, 23W11751, 23Q11754, 24U11012, 24S11013, 24X11016	Sanger sequencing (rs1799853, rs1057910); allelic discrimination PCR
*CYP1A2*	*1C, *1F	23Q15562, 23X15564, 23P15568, 23R15570	Allelic discrimination PCR
*CYP3A4*	*22	23R15570	Sanger sequencing
*CYP3A5*	*3, *7	23Q15559, 23V15565	Allelic discrimination PCR
*DPYD*	rs3918290 (*2A), rs75017182 + rs56038477 (*HapB3*), rs67376798 (*D949V*), rs55886062 (*13)	23U15557, 23S15558, 23U15560, 23U11752, 23P11746, 23W11748, 24R11022, 24P11023, 24W11025	Sanger sequencing; allelic discrimination PCR
*UGT1A1*	*28, *37, c.1220_1221insG	23U15557, 23S15558, 23T11744, 23U11749, 23U15560, 23U11752, 24V11017, 24T11018, 24R11019, 24V11020	STR analysis of (TA)n promoter repeat; Sanger sequencing; whole-exome sequencing
*TPMT*	*2, *3A, *3B, *3C	23T15566, 23R15567, 23Y15569	Allelic discrimination PCR
*NUDT15*	*3	23R15567, 23Y15569	Allelic discrimination PCR
*RYR1*	c.7858C>T (p.Gln2620*)	24S11027	Whole-exome sequencing

**Table 3:** Clinical categories, genes tested, and concordance results in the validation cohort.

Clinical Category	N (%)	Genes Tested	Sample IDs
Oncology (fluoropyrimidines, irinotecan, tamoxifen)	16 (39%)	*DPYD*, *UGT1A1*, *CYP2D6*	23U15557, 23S15558, 23U15560, 23T11744, 23U11749, 23U11746, 23W11748, 23R15570 *, 23U11752, 24R11022, 24P11023, 24W11025, 24V11017, 24T11018, 24R11019, 24V11020
Psychiatry (psychotropic therapy optimization)	10 (24%)	*CYP2D6*, *CYP2C19*, *CYP2C9*, *CYP1A2*, *CYP3A4*	23V15555, 23Q15562, 23Z15563, 23R15570 *, 23V15551, 23T15552, 23R15553, 23P15554, 23P15568, 23X15564
Neurology (siponimod therapy)	7 (17%)	*CYP2C9*	23R11745, 23Z11738, 23Q11740, 23W11751, 24U11012, 24S11013, 24X11016
Gastroenterology (thiopurines)	3 (7%)	*TPMT*, *NUDT15*	23T15566, 23R15567, 23Y15569
Transplantation (tacrolimus therapy)	2 (5%)	*CYP3A5*	23Q15559, 23V15565
Other individual cases	4 (10%)	*CYP2D6*, *CYP2C19*, *CYP2C9*, *RYR1*	23Y11750, 23Q11754, 23V11743, 24S11027

* Sample 23R15570 appears in both the “Oncology” and “Psychiatry” categories, as the diagnostic request included the evaluation of variants affecting both tamoxifen and psychotropic drug metabolism.

**Table 4:** Comparison of targeted NGS-based pharmacogenomic assays.

Panel Name	Total Genes	Capture Strategy	Coverage Depth (mean)	Structural Variant (SV) & CNV Detection	Key Validation Metrics
Action PharmaKitDx	335	Exons, UTRs, intronic boundaries + direct haplotype capture	582×–1880×	Yes (*CYP2D6* CNVs, hybrids, *HLA*)	100% sensitivity; 98% GeT-RM concordance
ClinPharmSeq [12]	59	Exon-centered + custom polymorphic loci	274×	Yes (via PyPGx)	96.3% WGS concordance
PGRNseq [19]	84	Exons + UTRs	200×–496×	Limited (requires specialized secondary pipelines)	>99% dataset concordance
ADME Panel [4]	340	Exons + regulatory regions	N/A	Limited to common SVs	>99% concordance

## Data Availability

The data presented in this study are available on request from the corresponding author. The data are not publicly available due to ethical, legal, and data protection restrictions. The minimal dataset supporting the conclusions of this article is available from the corresponding author upon reasonable request and subject to institutional approval and data-sharing agreements.

## Supplementary Materials

The following supporting information can be downloaded at https://www.mdpi.com/article/10.3390/ph19040568/s1, Table S1: Filtered rare variants.

## Abbreviations

The following abbreviations are used in this manuscript:

ADME	Absorption, Distribution, Metabolism, and Excretion
AEMPS	Agencia Española de Medicamentos y Productos Sanitarios
CNV	Copy Number Variation
CPNDS	Canadian Pharmacogenomics Network for Drug Safety
CPIC	Clinical Pharmacogenetics Implementation Consortium
DPWG	Dutch Pharmacogenetics Working Group
EMA	European Medicines Agency
FDA	U.S. Food and Drug Administration
GeT-RM	Genetic Testing Reference Materials Coordination Program
HLA	Human Leukocyte Antigen
IVDD	In Vitro Diagnostic Directive
NGS	Next-Generation Sequencing
NPA	Negative Percent Agreement
PCR	Polymerase Chain Reaction
PGx	Pharmacogenomics
PPA	Positive Percent Agreement
PPV	Positive Predictive Value
Q30	Phred Quality Score ≥30
SNV	Single Nucleotide Variant
STR	Short Tandem Repeat
SV	Structural Variant
UTR	Untranslated Region
WES	Whole-Exome Sequencing
WGS	Whole-Genome Sequencing

## Appendix A. Supplementary Methods

### Appendix A.1. Target Enrichment and Capture Design

To balance comprehensive pharmacogenomic coverage with sequencing efficiency, the Action PharmaKitDx assay uses a mixed capture strategy combining three complementary approaches. This design ensures high-resolution analysis in core genes while enabling the detection of targeted variants across an extended pharmacogenomic landscape.

#### Appendix A.1.1. Full Coding Regions with Extended Intronic and Regulatory Coverage (23 Genes)

- All coding exons
- ±50 bp of flanking intronic sequence
- Selected deep intronic regions containing known pathogenic variants
- Relevant 5′ and 3′ untranslated regions (UTRs)

This strategy maximizes detection of both common and rare functional variants. Genes captured under this strategy include *ABCB1*, *CACNA1S*, *CYP1A2*, *CYP2B6*, *CYP2C19*, *CYP2C8*, *CYP2C9*, *CYP3A4*, *DPYD*, *NUDT15*, *RYR1*, *SLCO1B1*, *TPMT*, *UGT1A1*, and additional UGT family members, as well as *VKORC1*.

#### Appendix A.1.2. Direct Haplotype Capture for Highly Polymorphic Genes (6 Genes)

For genes where haplotype or allele-level resolution is essential but SNV-based reconstruction is unreliable, the panel uses direct haplotype capture with custom probe sets. This strategy allows accurate classification without relying on individual SNV annotation.

Genes analyzed with this approach:

- CYP2D6
- HLA-B, HLA-C, HLA-DRB1, HLA-E, HLA-G

This ensures reliable detection of copy-number variations, hybrid alleles, and highly polymorphic HLA haplotypes.

#### Appendix A.1.3. Targeted Capture of Clinically Relevant Loci (307 Genes)

For an additional 307 pharmacogenes, the panel selectively captures:

- Coding exons and selected noncoding regions containing well-established pharmacogenetic variants
- Specific loci documented in curated databases or medical literature

This approach focuses sequencing depth on variants with known or potential clinical relevance while maintaining broad genomic scope.

#### Appendix A.1.4. Total Gene Content

In total, the panel evaluates 335 pharmacogenes, encompassing drug-metabolizing enzymes, transporters, receptors, immune response genes, and genes associated with adverse drug reactions. The complete list of genes included in the panel is provided in list (A1).

*ABCB1*, *ABCB11*, *ABCB4*, *ABCB7*, *ABCC1*, *ABCC2*, *ABCC3*, *ABCC4*, *ABCC5*, *ABCC6*, *ABCC8*, *ABCC9*, *ABCG1*, *ABCG2*, *ACE*, *ACKR1*, *ACYP2*, *ADA*, *ADD1*, *ADGRB3*, *ADGRL2*, *ADH1A*, *ADH1B*, *ADH1C*, *ADORA2A*, *ADRB1*, *ADRB2*, *AFF3*, *AHR*, *AKR1E2*, *ALDH1A1*, *ALDH2*, *ALOX5*, *ALPL*, *AMPD1*, *ANKK1*, *APOA5*, *APOE*, *ARL14*, *ATIC*, *ATP7A*, *BDNF*, *BMP2*, *BMP7*, *BRINP1*, *BTG1*, *C11orf65*, *C1orf167*, *C8orf34*, *CACNA1S*, *CADM2*, *CALU*, *CARD8*, *CBR3*, *CCDC179*, *CCHCR1*, *CCR5*, *CD2*, *CD226*, *CD69*, *CD80*, *CD84*, *CD86*, *CDA*, *CEP72*, *CES1*, *CES2*, *CETP*, *CHRNA3*, *CHRNB2*, *CHST7*, *CHUK*, *CLEC2D*, *CLSTN2*, *CNTN5*, *COL22A1*, *COMT*, *COQ2*, *CREB1*, *CRHR1*, *CRHR2*, *CRP*, *CSMD1*, *CSMD3*, *CUL1*, *CYP17A1*, *CYP19A1*, *CYP1A1*, *CYP1A2*, *CYP1B1*, *CYP2A13*, *CYP2A6*, *CYP2B6*, *CYP2C18*, *CYP2C19*, *CYP2C8*, *CYP2C9*, *CYP2D6*, *CYP2E1*, *CYP2F1*, *CYP2J2*, *CYP2S1*, *CYP3A4*, *CYP3A43*, *CYP3A5*, *CYP3A7*, *CYP4B1*, *CYP4F2*, *DCK*, *DHFR*, *DHODH*, *DLG2*, *DOCK1*, *DPP6*, *DPYD*, *DRD1*, *DRD2*, *DRD3*, *DRD4*, *DYNC2H1*, *EGF*, *EGLN3*, *EMCN*, *ENOX1*, *EPHX1*, *ERCC1*, *ESR1*, *ESR2*, *ETV1*, *EYA4*, *F5*, *FAAH*, *FAR1*, *FCGR2A*, *FCGR2B*, *FCGR3A*, *FDPS*, *FKBP5*, *FLT3*, *FMO2*, *FMO3*, *FOXP3*, *FPGS*, *FTO*, *G6PD*, *GABBR2*, *GALNT18*, *GATM*, *GFRA1*, *GFRA2*, *GGCX*, *GGH*, *GNB3*, *GP1BA*, *GRIK4*, *GSTM1*, *GSTP1*, *GSTT1*, *HAS3*, *HLA-A*, *HLA-B*, *HLA-C*, *HLA-DRB1*, *HLA-E*, *HLA-G*, *HMGCR*, *HTR1A*, *HTR2A*, *HTR2C*, *HTR3A*, *IFNG*, *IFNGR2*, *IFNL3*, *IFNL4*, *IGHM*, *IKBKB*, *IL10*, *IL12B*, *IL17A*, *IL17F*, *IL18*, *IL1B*, *IL1R1*, *IL2*, *IL4R*, *IL6*, *IL6R*, *IRAK3*, *ITGB7*, *ITPA*, *KCNE1*, *KCNH2*, *KCNIP1*, *KCNJ11*, *KIAA0391*, *KIF6*, *KLRC1*, *KLRD1*, *LMO3*, *LMX1A*, *LPA*, *LRPAP1*, *LRRC55*, *LTC4S*, *MAP2K6*, *MAP3K1*, *MAP3K14*, *MAP3K7*, *MAPK14*, *MAPKAPK2*, *MAPT*, *MC4R*, *MED15*, *MICA*, *MMP20*, *MRPL36*, *MS4A1*, *MTHFD1*, *MTHFR*, *MTR*, *MTRR*, *MYD88*, *NAT1*, *NAT2*, *NAV2*, *NEBL*, *NEDD4L*, *NFIB*, *NFKBIA*, *NFKBIB*, *NFKBIE*, *NLRP3*, *NQO1*, *NR1I2*, *NR2F2*, *NR3C1*, *NT5C2*, *NTRK2*, *NUBPL*, *NUDT15*, *OPRD1*, *OPRM1*, *OR2B11*, *OSR1*, *P2RY1*, *P2RY12*, *PDZD2*, *PLA2G4A*, *PNPLA3*, *PRELID3B*, *PRKCA*, *PRSS53*, *PTGFR*, *PTGIS*, *PTGS1*, *PTGS2*, *PTPRC*, *PTPRM*, *QKI*, *RAD51B*, *RHOBTB1*, *RPS6KA5*, *RYR1*, *SCN1A*, *SCN5A*, *SCNN1A*, *SEMA3C*, *SHMT1*, *SLC15A1*, *SLC15A2*, *SLC16A7*, *SLC19A1*, *SLC22A1*, *SLC22A11*, *SLC22A2*, *SLC22A6*, *SLC28A3*, *SLC46A1*, *SLC6A4*, *SLC9A7*, *SLCO1A2*, *SLCO1B1*, *SLCO1B3*, *SLCO1C1*, *SLCO2B1*, *SOD2*, *SP3*, *SRY*, *STAT4*, *STRBP*, *SULT1A1*, *SULT1A2*, *SYNM*, *TANC1*, *TBXAS1*, *TCF7L2*, *TEC*, *TGFB1*, *TGFBR2*, *TLR1*, *TLR10*, *TLR2*, *TLR4*, *TLR5*, *TNF*, *TNFAIP3*, *TNFRSF1A*, *TNFRSF1B*, *TNFSF13B*, *TP53*, *TPMT*, *TRAF1*, *TUBB2A*, *TYMS*, *UGT1A1*, *UGT1A10*, *UGT1A3*, *UGT1A4*, *UGT1A5*, *UGT1A6*, *UGT1A7*, *UGT1A8*, *UGT1A9*, *UGT2B10*, *UGT2B15*, *UGT2B17*, *UGT2B7*, *UMPS*, *VDR*, *VKORC1*, *WDR27*, *XPC*, *XRCC1*, *YEATS4*, *ZNF595*. (A1)

### Appendix A.2. Pharmacogenetic Alleles Included in the Panel

The Action PharmaKitDx platform incorporates a curated set of pharmacogenetically relevant alleles and haplotypes that are automatically inferred by a dedicated bioinformatic algorithm. Variant interpretation relies on a periodically updated allele database maintained by the clinical team at Health in Code, integrating information from international pharmacogenetic consortia, peer-reviewed literature, and population databases.

For each sample, the algorithm evaluates the detected SNVs, indels, and—in selected loci—structural or copy-number changes, assigning haplotypes according to established star allele nomenclatures. The system provides automated diplotype calls for a broad range of clinically relevant pharmacogenes, including but not limited to genes involved in drug metabolism (e.g., CYP family), drug transport (e.g., ABC transporters, *SLCO1B1*), immune-mediated toxicity (e.g., HLA alleles), and pharmacodynamic mechanisms (e.g., *RYR1*).

A list of alleles that can be automatically inferred by the algorithm is provided in list (A2):

*ABCC3 [rs1051640]* *ABCG2 [rs2231142]* *CACNA1S [c.1589G>A/p.Arg530His*, *c.1598G>A/p.Arg533His]* *CALU [c.606+133A>G (rs339097)]* *COMT [c.615+310C>T (rs4646316)]* *CYP1A2 [*1A*, **1C*, **1D*, **1F*, **1K*, **1L*, **2*, **3*, **4*, **5*, **6*, **7*, **8*, **11*, **15*, **16]* *CYP2B6 [*2*, **3*, **4*, **5*, **6*, **7*, **8*, **9*, **11*, **12*, **13*, **14*, **15*, **16*, **18*, **19*, **20*, **21*, **22*, **26*, **27*, **28]* *CYP2C18 [rs12777823]* *CYP2C19 [*2A*, **2B*, **3*, **4*, **5*, **6*, **7*, **8*, **9*, **10*, **12*, **13*, **14*, **15*, **17]* *CYP2C9 [*2*, **3*, **4*, **5*, **6*, **9*, **10*, **11*, **12*, **13*, **15*, **16*, **25]* *CYP2D6 [*2*, **3*, **4*, **5*, **6*, **7*, **8*, **9*, **10A*, **11*, **12*, **14A*, **14B*, **15*, **17*, **18*, **19*, **20*, **21A*, **29*, **38*, **40*, **41*, **42*, **44*, **56A*, **56B*, **64*, *x2*, *xN*, *hyb]* *CYP3A4 [*1B*, **2*, **3*, **4*, **5*, **6*, **7*, **8*, **10*, **11*, **12*, **13*, **14*, **15A*, **15B*, **16*, **17*, **18*, **19*, **20*, **22]* *CYP3A5 [*1A*, **2*, **3B*, **3C*, **3D*, **3F*, **3G*, **3K*, **3L*, **4*, **5*, **6*, **7*, **8*, **9]* *CYP4F2 [*1*, **2*, **3]* *DPYD [c.1024G>A (rs183385770)*, *c.1057C>T (rs143154602)*, *c.1129-5923C>G*, *c.1236G>A (HapB3)*, *c.1156G>T (*12)*, *c.1314T>G (rs186169810)*, *c.1484A>G (rs111858276)*, *c.1679T>G (*13)*, *c.1774C>T (rs59086055)*, *c.1775G>A (rs138616379)*, *c.1777G>A (rs145773863)*, *c.1898delC (*3)*, *c.1905+1G>A (*2A)*, *c.2021G>A (rs137999090)*, *c.2279C>T (rs112766203)*, *c.2639G>T (rs55674432)*, *c.2656C>T (rs147545709)*, *c.2846A>T (rs67376798)*, *c.2872A>G (rs141044036)*, *c.2933A>G (rs72547601)*, *c.295_298delTCAT (*7)*, *c.2983G>T (*10)*, *c.557A>G (rs115232898)*, *c.601A>C (rs72549308)*, *c.61C>T (rs72549310)*, *c.632A>G (rs72549307)*, *c.703C>T (*8)*, *c.868A>G (rs146356975)]* *F5 [Factor V Leiden]* *G6PD [202G>A_376A>G_1264C>G*, *A*, *A- 202A_376G*, Aachen, Abeno, Acrokorinthos, Alhambra, Amazonia, Amiens, Amsterdam, Anadia, Ananindeua, Andalus, Arakawa, Asahi, Asahikawa, Aures, Aveiro, B (wildtype), Bajo Maumere, Bangkok, Bangkok Noi, Bao Loc, Bari, Belem, Beverly Hills, Genova, Iwate, Niigata, Yamaguchi, Brighton, Buenos Aires, Cairo, Calvo Mackenna, Campinas, Canton, Taiwan-Hakka, Gifu-like, Agrigento-like, Cassano, Chatham, Chikugo, Chinese-1, Chinese-5, Cincinnati, Cleveland Corum, Clinic, Coimbra Shunde, Cosenza, Costanzo, Covao do Lobo, Crispim, Dagua, Durham, Farroupilha, Figuera da Foz, Flores, Fukaya, Fushan, G6PD A- 680T_376G, G6PD A- 968C_376G, G6PDNice, Gaohe, Georgia, Gidra, Gond, Guadalajara, Guangzhou, Haikou, Hammersmith, Harilaou, Harima, Hartford, Hechi, Hermoupolis, Honiara, Ierapetra, Ilesha, Insuli, Iowa, Walter Reed, Springfield, Iwatsuki, Japan, Shinagawa, Kaiping, Anant, Dhon, Sapporo-like, Wosera, Kalyan-Kerala, Jamnaga, Rohini, Kambos, Kamiube, Keelung, Kamogawa, Kawasaki, Kozukata, Krakow, La Jolla, Lages, Lagosanto, Laibin, Lille, Liuzhou, Loma Linda, Ludhiana, Lynwood, Madrid, Mahidol, Malaga, Manhattan, Mediterranean Haplotype, Mediterranean, Dallas, Panama‚ Sassari, Cagliari, Birmingham, Metaponto, Mexico City, Miaoli, Minnesota, Marion, Gastonia, LeJeune, Mira d’Aire, Mizushima, Montalbano, Montpellier, Mt Sinai, Munich, Murcia Oristano, Musashino, Namouru, Nankang, Nanning, Naone, Nara, Nashville, Anaheim, Portici, Neapolis, Nilgiri, No name, North Dallas, Olomouc, Omiya, Orissa, Osaka, Palestrina, Papua, Partenope, Pawnee, Pedoplis-Ckaro, Piotrkow, Plymouth, Praha, Puerto Limon, Quing Yan, Radlowo, Rehevot, Rignano, Riley, Riverside, Roubaix, S. Antioco, Salerno Pyrgos, Santa Maria, Santiago, Santiago de Cuba, Morioka, Sao Borja, Seattle, Lodi, Modena, Ferrara II, Athens-like, Seoul, Serres, Shenzen, Shinshu, Sibari, Sierra Leone, Sinnai, Songklanagarind, Split, Stonybrook, Sugao, Sumare, Sunderland, Surabaya, Suwalki, Swansea, Taipei‚ Chinese-3, Telti/Kobe, Tenri, Tokyo, Fukushima, Toledo, Tomah, Tondela, Torun, Tsukui, Ube Konan, Union, Maewo, Chinese-2, Kalo, Urayasu, Utrecht, Valladolid, Vancouver, Vanua Lava, Viangchan, Jammu, Villeurbanne, Volendam, Wayne, West Virginia, Wexham, Wisconsin, Yunan] *GGCX [c.2084+45G>C (rs11676382))]* *HLA-A [c.*66A>T (rs1061235-T) (*31:01)]* *HLA-B [base de datos IMGT/HLA v 3.12 compuesta 2932 alelos]* *IFNL4 [c.151-152G>A (rs12979860)]* *NAT1 [*4*, **5*, **11*, **11C*, **14*, **15*, **17*, **19A*, **19B*, **22*, **23*, **27*, **30]* *NAT2 [*4*, **5A*, **5E*, **6A*, **6J*, **7A*, **7D*, **10*, **12D*, **14A*, **14D*, **14F*, **17*, **18*, **19]* *NUDT15 [*2*, **3*, **4*, **5*, **6*, **7*, **8*, **9*, **10*, **11*, **12*, **13*, **14*, **15*, **16*, **17*, **18*, **19]* *RYR1 [p.Ala2350Thr*, *p.Ala2428Thr*, *p.Arg163Cys*, *p.Arg163Leu*, *p.Arg2163Cys*, *p.Arg2163His*, *p.Arg2336His*, *p.Arg2355Trp*, *p.Arg2435His*, *p.Arg2452Trp*, *p.Arg2454Cys*, *p.Arg2454His*, *p.Arg2458Cys*, *p.Arg2458Leu*, *p.Arg2508Cys*, *p.Arg2508Gly*, *p.Arg2508His*, *p.Arg328Trp*, *p.Arg401Gly*, *p.Arg44Cys*, *p.Arg4861His*, *p.Arg530His*, *p.Arg533His*, *p.Arg533Ser*, *p.Arg552Trp*, *p.Arg614Cys*, *p.Arg614Leu*, *p.Glu2348del*, *p.Glu3104Lys*, *p.Gly2375Ala*, *p.Gly2434Arg*, *p.Gly248Arg*, *p.Gly341Arg*, *p.Gly3990Val*, *p.His4833Tyr*, *p.Ile403Met*, *p.Ile4898Thr*, *p.Leu4838Val*, *p.Thr2206Arg*, *p.Thr2206Met*, *p.Thr4826Ile*, *p.Tyr4796Cys*, *p.Tyr522Ser*, *p.Val2168Met*, *p.Val4849Ile]* *SLC28A3 [c.1381C>T*, *(rs7853758)]* *SLCO1B1 [*1B*, **2*, **3*, **4*, **5*, **6*, **7*, **8*, **9*, **11*, **13*, **14*, **15*, **16*, **17*, **18*, **21*, **31]* *TPMT [*1*, **2*, **3A*, **3B*, **3C*, **3D*, **4*, **8*, **24]* *UGT1A1 [*1*, **27*, **28*, **36*, **37*, **6*, **60*, **93]* *UGT1A6 [*1*, **2*, **3A*, **4A*, **4b*, **5]* *VKORC1 [c.-1639G>A (rs9923231)]*. (A2)

### Appendix A.3. Analytical Performance Metrics

The analytical performance of the Action PharmaKitDx panel was evaluated using standard metrics for genotyping assays. Below are the definitions and formulas applied:

Analytical accuracy: proportion of correctly identified genotypes compared to reference materials.

(A3) $$Accuracy\left(\%\right)=\frac{True\;Positive+True\;Negative}{True\;Positive+False\;Positive+True\;Negative+False\;Negative}\times 100$$

Analytical sensitivity (positive percent agreement, PPA): proportion of true positive variants correctly detected.

(A4) $$PPA\left(\%\right)=\frac{True\;Positive}{True\;Positive+False\;Negative}\times 100$$

Analytical specificity (negative percent agreement, NPA): proportion of true negative positions correctly identified.

(A5) $$NPA\left(\%\right)=\frac{True\;Negative}{False\;Positive+True\;Negative}\times 100$$

Precision (positive predictive value, PPV): proportion of variant calls that are true positives.

(A6) $$PPV\left(\%\right)=\frac{True\;Positive}{True\;Positive+False\;Positive}\times 100$$

Repeatability, reproducibility and robustness: consistency of results within the same sequencing run (repeatability), across different sequencing runs (reproducibility), and across different sequencing instruments.

(A7) $$Repeatability\left(\%\right)=\frac{Concordant\;calls\;Intra-Run}{Total\;calls\;Intra-Run}\times 100\;\;Reproducibility\left(\%\right)=\frac{Concordant\;calls\;Inter-Run}{Total\;calls\;Inter-Run}\times 100\;\;Robustness\left(\%\right)=\frac{Concordant\;calls\;Inter-Instrument}{Total\;calls\;Inter-Instrument}\times 100$$

### Appendix A.4. Rare Variant Filtering and Prioritization Workflow

A multi-step filtering pipeline was applied to the full set of annotated variants to identify high-confidence rare variants of potential clinical relevance. The workflow integrated quality metrics, population allele frequencies, genomic context, in silico predictions, and ClinVar classifications. All filtering steps were implemented programmatically to ensure reproducibility.

#### Appendix A.4.1. Quality-Based Filtering

Variants were first required to meet minimal sequencing quality standards:

- Depth of coverage ≥ 30
- Base quality ≥ 100
- Allele fraction thresholds adapted to zygosity:
  a. Heterozygous variants ≥ 0.25
  b. Homozygous variants ≥ 0.80
  c. Variants with undefined zygosity were conservatively required to meet ≥ 0.25

Variants failing any of these conditions were excluded from downstream evaluation.

#### Appendix A.4.2. Population Frequency Filtering

To retain only rare or ultra-rare alleles, population allele frequencies were examined across the following databases:

- gnomAD
- 1000 Genomes
- 5000 Exomes
- dbSNP frequency annotations

Variants were retained only when all available population frequency values were <1%, or when no population frequency data were available (classified as ultra-rare).

#### Appendix A.4.3. Functional Annotation Filtering

Variants were then evaluated for their genomic context and potential functional impact. Variants were kept if they fulfilled at least one of the following criteria:

- Located in coding regions or exons
- Caused a non-synonymous amino acid change, including missense, nonsense, frameshift, indels, or disruptions affecting the reading frame
- Annotated as splicing-relevant, either through:
  a. Explicit splicing flags in the annotation, or
  b. NNSPLICE predictions indicating ≥10% reduction in splice-site strength

Synonymous variants were excluded unless supported by splicing predictions.

#### Appendix A.4.4. Pathogenicity Predictor Filtering

To prioritize variants with in silico evidence of deleteriousness, these complementary tools were selected for their established utility in predicting disruptions to protein function in pharmacogenetic contexts. At least one of the following had to apply:

- DANN score ≥ 0.9
- FATHMM coding-group classification containing “damaging” or “deleterious”
- MutationTaster prediction = “D” (disease causing)

Variants lacking predictor data were not excluded unless previously removed in an earlier step.

#### Appendix A.4.5. ClinVar-Based Filtering

ClinVar annotations were used to integrate clinical evidence:

- Variants classified as pathogenic, likely pathogenic, conflicting, or VUS were retained.
- Variants labelled benign or likely benign were excluded unless at least one in silico predictor suggested deleteriousness (as defined above).
- Variants with no ClinVar annotation passed this step by default.

#### Appendix A.4.6. Final Variant Set

Only variants passing all filtering stages were included in the final prioritized list. Across the full dataset, this workflow identified 36 rare variants with potential functional and clinical relevance.

## Appendix B. Supplementary Results

### Appendix B.2. Analytical Performance

**Table A1:** Detailed analytical performance counts across reference samples and sequencing workflows.

Workflow	Sample	Replicates	True Positives (TP)	True Negatives (TN)	False Positives (FP)	False Negatives (FN)	Mean Accuracy (%)
MiSeq (Manual)	NA12878	3	109	703	0	0	100
	NA12891	3	109	703	0	0	100
	NA12892	3	104–107	705	0	0 (Rep 1), 3 (Rep 2,3)	99.6
MiSeq (Automated)	NA12878	3	109	703	0	0	100
	NA12891	3	109	703	0	0	100
	NA12892	3	107	705	0	0	100
NextSeq 500/550	NA12878	3	109	703	0	0	100
	NA12891	3	108–109	703	0	0 (Rep 1,2), 1 (Rep 3)	99.9
	NA12892	3	106–107	705	0	0 (Rep 1,2), 1 (Rep 3)	99.9
NextSeq 1000/2000	NA12878	3	109	703	0	0	100
	NA12891	3	109	703	0	0	100
	NA12892	3	106–107	705	0	0 (Rep 1,2), 1 (Rep 3)	99.9

(Note: Ranges in TP indicate expected biological variation in the reference consensus or minor depth fluctuations between specific technical replicates).
